# Long-Term Health Outcomes in Offspring Born to Women with Diabetes in Pregnancy

**DOI:** 10.1007/s11892-014-0489-x

**Published:** 2014-03-25

**Authors:** Abigail Fraser, Debbie A. Lawlor

**Affiliations:** 1 MRC Integrative Epidemiology Unit, University of Bristol, Oakfield House, Oakfield Grove, Bristol, BS8 2BN UK; 2 School of Social and Community Medicine, University of Bristol, Oakfield House, Oakfield Grove, Bristol, BS8 2BN UK

**Keywords:** Pregnancy diabetes, Developmental overnutrition, Cognitive ability, Adiposity, Cardiometabolic health, Epigenetics, Long-term health outcomes, Offspring

## Abstract

In this review, we critically assess recent evidence from human studies regarding the potential implications of exposure to maternal diabetes in-utero for long-term adiposity, cardiometabolic outcomes, and cognitive ability of the offspring. Evidence supports a direct causal role for exposure to maternal diabetes in utero in determining offspring long-term greater adiposity and adverse cardiometabolic health. Although a majority of observational studies report associations of exposure to maternal pregnancy diabetes with lower cognitive ability, there is also evidence supporting an opposite ‘protective’ intrauterine effect of exposure to maternal pregnancy diabetes on offspring cognitive ability. Epigenetic modification has been suggested as a mediator on the pathways from maternal pregnancy diabetes to long-term offspring outcomes and several recent studies that are reviewed here lend some support to this notion, but research in this area is still too novel to be conclusive.

## Introduction

Diabetes in pregnancy can be 1 of the following 3 types of diabetes: pre-existing type 1 diabetes (present before pregnancy), pre-existing type 2 diabetes, or gestational diabetes, (ie, diabetes with onset or first diagnosis in pregnancy). Increases in the prevalence of all 3 types of pregnancy diabetes have been noted in recent years [1–4], with virtually identical US and UK estimates suggesting that gestational diabetes accounts for the majority of diabetic pregnancies (~87 %) and, type 1 diabetes for an additional 7 % and type 2 diabetes for 5 % [5, 6]. Although the US National Institute of Health recently estimated the prevalence of gestational diabetes to be 5 %–6 % [7], other studies have yielded estimates as high as 14 % [8, 9].

Perinatal complications of a diabetic pregnancy include macrosomia, hypoglycemia, respiratory distress syndrome, polycythemia, hyperbilirubinemia, cardiomyopathy, congenital abnormalities, and sudden infant death. These have been extensively reviewed elsewhere in recent years [10], including in this Journal [11], and are not the focus here. The purpose of the current review is to summarize and discuss recent evidence from human studies regarding the potential implications of exposure to maternal pregnancy diabetes in utero for offspring long-term greater adiposity and adverse cardiometabolic outcomes, and cognitive ability, with the outcomes examined reflecting the latest and most extensive work on long-term offspring health outcomes following in-utero exposure to maternal diabetes. We will consider each of these outcomes separately, outlining potential pathways and underlying mechanisms linking maternal pregnancy diabetes with offspring outcomes, including potential mediation by epigenetic processes, namely DNA methylation. These will anchor our review of the literature as we critically assess whether it supports a direct causal relationship between maternal pregnancy diabetes and these long-term offspring health outcomes, or not.

**Fig. 1:**
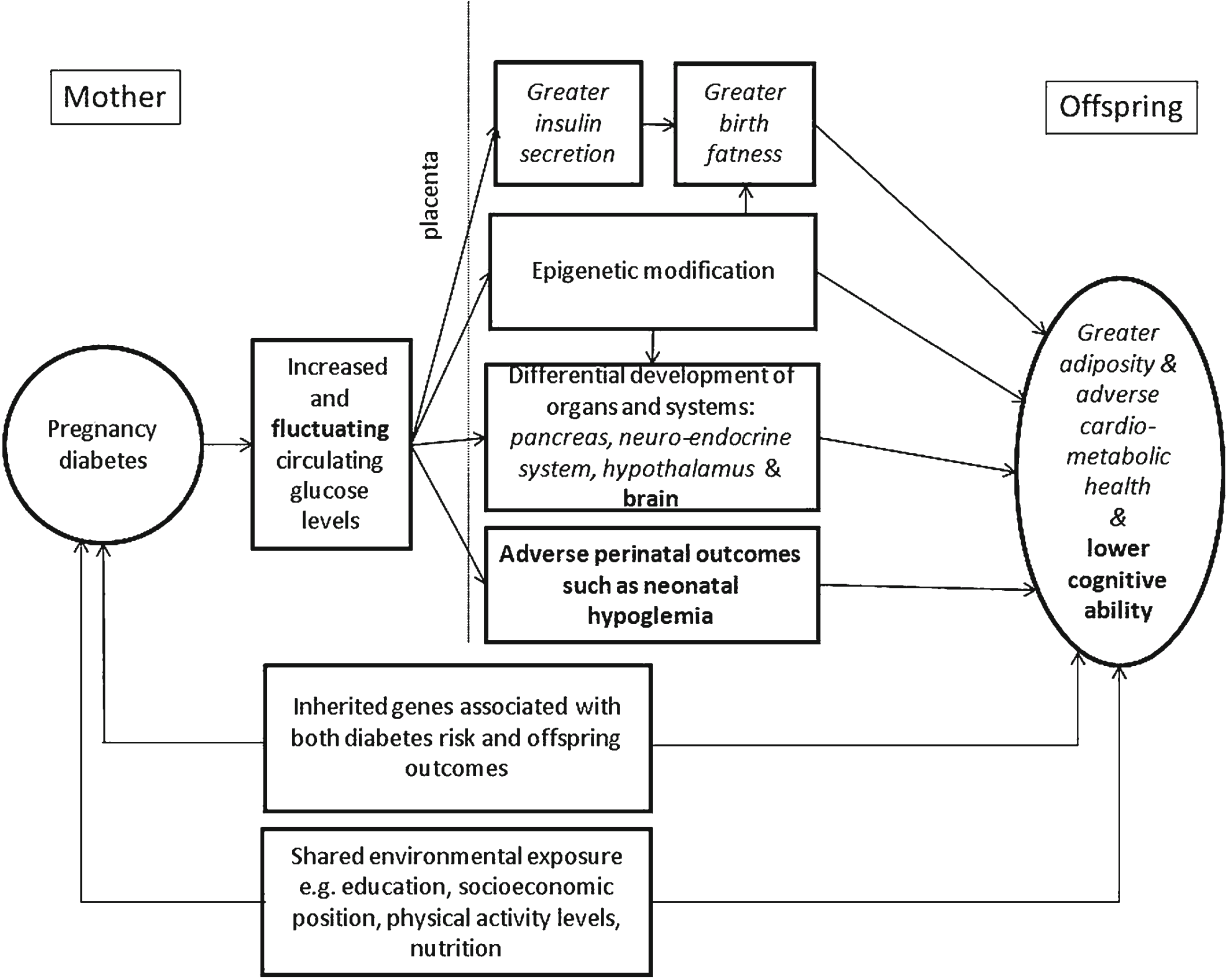
Schematic representation of potential pathways linking maternal diabetes in pregnancy with long- term offspring greater adiposity, adverse cardiometabolic health and lower cognitive ability. The figure is illustrative of the focus of this article and does not aim to show all possible relationships between the characteristics that are depicted. Pathways specific to offspring adiposity and cardiometabolic health are given in *italics* and pathways specific to cognitive ability in **bold** with common pathways in standard font. Adapted from Lawlor DA. The Society for Social Medicine John Pemberton lecture 2011. Developmental overnutrition—an old hypothesis with new importance? Int J Epidemiol. 2013;42:7–29 [14•]; and Fraser A, Almqvist C, Larsson H, Långström N, Lawlor D. Maternal diabetes in pregnancy and offspring cognitive ability: sibling study with 723,775 men from 579,857 families. Diabetologia. 2013;1–8 [47], which is published under the CC-BY license.)

Potential pathways linking maternal pregnancy diabetes and offspring long-term adiposity, cardiometabolic health, and cognitive ability are illustrated in Fig. 1. As can be seen, pathways depicting a direct effect of exposure to maternal diabetes in utero (as opposed to common genetic or environmental causes pathways), suggest that all diabetes types affect offspring outcomes via the same biological mechanisms, ie, the delivery of excess and/or fluctuating levels of nutrients to the fetus, which in turn influences fetal development and long-term outcomes. Associations between maternal glucose levels in pregnancy and neonatal outcomes including birth weight and adiposity have been shown to be graded across the entire distribution of glucose even in normoglycemic women, with no threshold effect [12••, 13]. It is likely that this is the case for long-term outcomes as well. However, women who have entered pregnancy with pre-existing diagnosed diabetes (type 1 or type 2) are more likely to have received counselling and monitoring and hence, have better controlled glucose levels than women diagnosed with gestational diabetes, ie, in the second or third trimester of pregnancy. Thus, offspring exposed to gestational diabetes as opposed to pre-existing type 1 or type 2 diabetes, may have been exposed to higher circulating levels of glucose at the earlier stages of pregnancy [14•].

## Offspring Adiposity and Cardiometabolic Health

Infants of diabetic mothers are often macrosomic or large for gestational age (LGA) [15–17]. This is due to increased delivery of glucose to the developing fetus. Glucose crosses the placenta while maternal insulin does not. As a result, fetal insulin production is increased, which in turn increases insulin mediated fetal fat deposition and skeletal growth [18•]. The observation that diabetic mothers have larger babies gave rise to the developmental overnutrition hypothesis, which extends this relationship both to maternal adiposity and to lasting effects on adiposity and related cardiometabolic measures that go beyond birth into childhood, adolescence, and even adulthood [14•]. Here we focus on the long-term consequences of maternal pregnancy diabetes, and readers are referred to 2 previous publications for further discussion of the evidence regarding long-term consequences of maternal adiposity, and gestational weight gain [14•, 18•], and for a summary of the evolution of the developmental overnutrition hypothesis [14•].

It is important to understand whether maternal diabetes (and indeed greater adiposity, which is not addressed here) is associated with lasting greater offspring adiposity and more adverse cardiometabolic health via intrauterine mechanisms given that developmental overnutrition could perpetuate the obesity epidemic across future generations. For this reason it is also important to identifying the exact nature of these mechanisms. According to the developmental overnutrition hypothesis female offspring of mothers with diabetes enter their own pregnancies more adipose, at greater risk of having diabetes in pregnancy, with consequences for their own offspring.

A growing number of reports based on data from prospective pregnancy cohorts support the developmental overnutrition hypothesis, for example [19, 20]. Interestingly, 2 recent systematic reviews [21, 22] suggested that the evidence supporting association between gestational diabetes [21], pregnancy diabetes [22], and greater offspring adiposity is still inconclusive. This conclusion was based on the reported attenuation of associations between pregnancy diabetes and offspring adiposity upon adjustment for maternal prepregnancy body mass index (BMI) in some studies. However, many of the studies included in these reviews were conducted in European and North American countries, in which screening for gestational diabetes is not universal and is only offered to women considered to be at high risk. This is likely to result in bias by indication [14•]. In other words, there will be more normal weight women with diabetes who are misclassified as nondiabetic because they will have been unlikely to have been offered a diagnostic oral glucose tolerance test than overweight or obese women. This means that adjustment for pre- or early pregnancy BMI results in over-attenuation of the association between pregnancy diabetes and offspring outcomes [14•].

Although observational studies do not in themselves provide proof for a role of direct intra-uterine mechanisms, a consistent body of evidence suggests that inheritance of genes predisposing to diabetes and/or shared behavior and other environmental exposures only partly explain associations of maternal pregnancy diabetes and offspring greater adiposity and associated adverse cardiometabolic health. Family based studies of the Pima Indians of Arizona, a population with a very high prevalence of obesity and type 2 diabetes, have yielded convincing evidence that exposure to diabetes in pregnancy per se results in greater mean body mass index (BMI), fasting glucose and insulin, and risk of type 2 diabetes in offspring (during childhood, adolescence, and early adulthood). This body of work has been reviewed in detail elsewhere [14•, 23•, 24]. Briefly, it has shown that offspring born to mothers who had diabetes during their pregnancy had greater mean body mass index (BMI), fasting glucose and insulin and risk of type 2 diabetes compared with either the offspring of mothers who developed diabetes later in their lives or those who never developed diabetes [24, 25]. Sibling studies are a powerful approach for determining causality as they inherently control for maternal genetic variation and any environmental exposures that have remained constant or very similar across pregnancies. Hence, in this context, a difference in the risk of obesity and type 2 diabetes between siblings is likely due, at least in part, to discordant exposure to maternal diabetes in-utero. The same study also found that within siblings there was no association of paternal diabetes around the time of pregnancy with these offspring outcomes [25]. Comparing the association of paternal and maternal diabetes with offspring outcomes is another way of exploring whether exposure to diabetes in utero is causally associated with offspring outcomes. If exposure to diabetes in utero directly affects the offspring the maternal-offspring association will be much stronger than the paternal-offspring association. However, associations driven by shared familial, social, genetic, and environmental factors are likely to produce similar maternal-paternal associations. Therefore, findings from the Pima sibling study suggest that, at least in a population at high risk for obesity and diabetes, intra-uterine mechanisms make an important contribution to the link between gestational diabetes and offspring greater adiposity, insulin resistance, and type 2 diabetes.

More recently, a large study of Swedish men included a within sibling analysis (280,866 men from 248,293 families) and found that the BMI of men whose mothers had diabetes during their pregnancy was on average 0.94 kg/m^2^ greater (95 % CI: 0.35, 1.52) than in their brothers born before their mother was diagnosed with diabetes [26]. There was no within-sibling association of maternal early pregnancy BMI in that study, and adjustment for it did not alter the diabetes-offspring BMI association. These findings suggest that in a European population with a much lower rate of pregnancy diabetes than the Pima Indians of Arizona, intra-uterine mechanisms contribute importantly to the link between maternal diabetes in pregnancy and later offspring adiposity and that this association is not strongly confounded by early pregnancy BMI.

## Offspring Cognitive Abilities

As already noted, maternal diabetes in pregnancy is associated with a number of neonatal complications and it has been postulated that these may, in turn, adversely affect offspring cognitive development [27, 28]. It is also possible that maternal diabetes in pregnancy affects fetal neurocognitive development and long-term cognitive ability through altered brain structure caused by in-utero exposure to a metabolic milieu, which incorporates high or fluctuating concentrations of glucose, and potentially ketonemia [29]. This hypothesized pathway is similar to the developmental overnutrition hypothesis but is concerned with cognitive development. Alternatively, pleiotropic effects of genes shared by mother and offspring that are related to both risk of diabetes and IQ could result in an association between pregnancy diabetes and offspring cognition. Finally, shared familial environmental exposures such as socioeconomic position, educational attainment, levels of physical activity and nutrition may result in associations between pregnancy diabetes and impaired offspring cognitive ability. These potential pathways are summarized in Fig. 1.

A number of studies have compared the cognitive abilities of offspring of mothers with pregnancy diabetes to offspring of mothers without pregnancy diabetes at different ages and using different measures of cognitive ability [28, 30–36]. Even though studies have reported lower scores in offspring of women with pregnancy diabetes on some indices, differences in overall IQ have been observed only for women with gestational diabetes and in younger ages [30, 34, 37]. Recent studies have examined associations between pregnancy diabetes and measures of educational attainment. Arguably, these are more significant outcomes as they have a direct impact on future educational and occupational outcomes. In a large Swedish record linkage study of 6397 offspring of mothers with pregnancy diabetes and ~1.3 children to mothers without pregnancy diabetes, maternal diabetes in pregnancy was associated with a greater risk of not completing compulsory schooling at the age of 16 (OR = 1.25; 95%CI: .10–1.43), and with a lower mean school mark [38]. Similarly, in the UK Avon Longitudinal Study of Parents and Children (ALSPAC), pre-existing diabetes (likely to diabetes type; *N* = 20 to 26 varying by outcome), gestational diabetes (*N* = 23 to 31), and, to a lesser extent, glycosuria (*N* = 160 to 240) were associated with lower offspring School Entry Assessment scores (at age 4), IQ (at age 8), and school results (GCSE results, at age 16) even when adjusting for potential confounders such as prepregnancy BMI, maternal smoking in pregnancy, parity, mode of delivery, maternal education, and social class [39]. In contrast to those 2 studies in populations with relatively low rates of pregnancy diabetes (0.5 %), in an Indian population characterized by higher rates of pregnancy diabetes (7 %), offspring of women with gestational diabetes (*N* = 32) scored higher on a variety of cognitive tests compared with offspring of women without gestational diabetes (*N* = 483) at a mean age of 9.7 years, though after adjusting for a range of potential confounders, differences were somewhat attenuated toward the null [40].

Smaller studies have reported correlations between indices of maternal metabolic control in women with and without pregnancy diabetes (eg, glucose, beta-hydroxybutyrate, free fatty acids, acetonuria), and offspring cognitive abilities in childhood. Here too, results have been mixed, with positive or null associations between different measures of metabolic control and different measures of cognitive ability found in the same study populations [27, 28, 41]. More recently, Nielsen et al reported negative associations between maternal pregnancy glycated hemoglobin (A1c) levels and offspring cognitive ability assessed at 18-20 years of age (*N* = 39) [42], as well as between pregnancy fasting blood glucose levels of insulin treated diabetic mothers and offspring cognitive ability assessed in early adulthood (*N* = 60) [43].

If associations between better maternal metabolic control in pregnancy and lower offspring cognitive ability are causal, this would be additional evidence supporting the need to identify and control glucose intolerance in pregnancy. However, observational epidemiologic studies may be affected by confounding, reverse causation and bias, and therefore, it is problematic to infer causality from their results. Two recent studies have employed different strategies to tackle these shortcomings with regard to associations between pregnancy diabetes and offspring cognitive abilities.

A Mendelian randomization approach uses genetic variants that are reliably associated with a modifiable exposure to make inferences about the causal effect of this exposure on an outcome of interest [44, 45]. This instrumental variable approach assumes that a genetic variant is associated with a trait of interest, here pregnancy diabetes; that the association between the variant and the trait is independent of unmeasured confounders and that the variant is associated with the outcome only through its association with the specific trait [45]. Thus, such an approach yields an unconfounded causal estimate of the association between exposure and outcome. Bonilla et al studied associations of maternal fasting glucose genetic risk scores and maternal type 2 diabetes genetic risk score with offspring IQ assessed at 8 years of age in the ALSPAC cohort [46]. Genetic risk scores combine data on multiple genetic variants that are known to be associated with a given trait and are used as they explain more of the variation in the trait than a single variant on its own. When these scores are then related to an outcome this provides a statistical test for whether that trait (with it, rather than the genes, being the risk factor of interest), is causally related to the outcome [44, 45]. In this study, authors found a *positive* association between maternal fasting glucose risk scores and offspring IQ, ie, a higher maternal risk score (indicative of higher fasting glucose) was associated with higher offspring IQ, but the confidence interval was wide and included the null value. A *positive* association was also found between maternal type 2 diabetes risk scores and offspring IQ even when adjusting for offspring’s own type 2 diabetes risk score; altough the confidence interval for this association was also wide, it did not include the null value. These findings suggest that maternal pregnancy diabetes and perhaps higher fasting glucose levels may result in greater offspring IQ in childhood.

In a sibling study using data from the Swedish population registers, we examined associations of maternal pregnancy diabetes with male offspring educational attainment at 16 years of age and IQ assessed at 18 years of age at the national conscription assessment. In nonsiblings, men whose mother had pregnancy diabetes had an IQ that was on average –1.36 points (95 % CI: –2.12, –0.60) lower than men whose mothers did not have diabetes in pregnancy even when adjusting for birth year, maternal age, education, and BMI in early pregnancy, parity as well as gestational age and birth weight. In comparison, there was no such association within sibships. In fact, siblings exposed to maternal diabetes in pregnancy had, on average, a higher IQ compared with their unexposed siblings: mean difference = 1.70; 95 % CI: –1.80, 5.21 (*p* value for difference between the 2 estimates 0.08). A similar pattern was found for school grades: in the whole population school grades of offspring of women with pregnancy diabetes were lower compared with offspring of women without diabetes in nonsiblings, even when adjusting for potential confounders, whilst associations were in the opposite direction with confidence intervals spanning the null value within sibships [47]. These results suggest that similarities shared by siblings and inherently controlled for when comparing siblings account for the associations between maternal pregnancy diabetes and lower cognitive ability observed in the cohort as a whole and between nonsiblings. In other words, findings suggest that a direct effect of exposure to a diabetic milieu and/or perinatal complications resulting from maternal diabetes (Fig. 1) are unlikely explanations for the associations between maternal diabetes in pregnancy and lower offspring cognitive abilities in the overall population.

Even as most observational studies report associations between exposure to maternal pregnancy diabetes, worse metabolic control, and lower offspring cognitive ability, there are now 3 different studies [40, 46, 47] with findings that support an opposite, positive effect of exposure to maternal pregnancy diabetes on long-term cognitive ability. The Swedish sibling study [47] found the expected association of maternal pregnancy diabetes with lower cognitive ability in the population as a whole but an analysis restricted to siblings discordant in their exposure to maternal pregnancy diabetes in utero suggested that exposure to maternal pregnancy diabetes was associated with higher cognitive ability, though statistical power was limited. These results point to shared environmental or genetics as drivers of the observed association between maternal pregnancy diabetes and lower cognitive ability in adolescence and early adulthood. At this time we are unaware of any existing evidence of genetic variants that are associated with both diabetes and cognitive ability (genetic pleiotropy) and hence, could explain the observed association of pregnancy diabetes with lower cognitive ability. Similarly, a Mendelian randomization study results are consistent with a protective effect of maternal pregnancy diabetes on fetal brain development [46], though, like the sibling comparison it too was hampered by limited statistical power.

### Mediation by Epigenetic Processes

Epigenetic modifications, such as DNA methylation, regulate gene expression without altering the underlying DNA sequence. They have been shown to occur in response to environmental stimuli [48], are common during mitosis and hence, during fetal development, and to be stable over time [49]. Hence, they are a plausible mediator of the effect of intrauterine exposures, such as exposure to maternal pregnancy diabetes, on offspring outcomes and are increasingly studied in this regard. In relation to the topic of this review most studies have examined the potential of epigenetic modification to influence future offspring greater adiposity and adverse cardiometabolic health than their cognitive function.

For epigenetic mechanisms to play an important part on the causal pathway between maternal pregnancy diabetes and offspring later adiposity, cardiometabolic and cognitive outcomes, there would need to be: (I) a causal effect of maternal diabetes on the fetal epigenome and (II) a causal effect of the specific fetal epigenome modifications resulting from pregnancy diabetes, on later offspring outcomes. To our knowledge to date no study has examined associations of this full chain (ie, the association of pregnancy diabetes with offspring epigenome and of it with later offspring outcomes). Therefore, here we first describe studies of: (I) maternal diabetes with fetal epigenome as the outcome and then of (II) associations of fetal epigenome as the exposure with later adiposity and cardiometabolic health. We then briefly discuss how causal epigenomic mediation might be tested. Whilst epigenetic mechanisms have been implicated in the etiology of cognitive function and disorders [50], we are unaware of any studies examining associations of fetal epigenome with cognitive outcomes later in life.

#### Maternal Pregnancy Diabetes and Fetal Epigenome

Bouchard et al reported finding no difference in mean DNA methylation levels of the leptin gene in placental tissue between 23 women who had experienced gestational diabetes and 25 age- and BMI-matched controls. However, in those with gestational diabetes, 2-hour postload glucose was inversely correlated with fetal-side placental tissue DNA methylation of the leptin gene, but positively correlated with this in maternal-side tissue [51]. In a second study from the same group, maternal 2-hour postload glucose was inversely associated with DNA methylation levels of the adiponectin gene on the fetal side of the placenta in 98 women [52]. In a third study, genome wide methylation profiles of placenta and cord blood samples of 30 women with gestational diabetes and 14 women without gestational diabetes assessed using the Infinium HumanMethylation450 BeadChips [53]. Authors found numerous differentially methylated genes (3271 and 3758 genes in placenta and cord blood, respectively, with 25 % overlap), though none reached the epigenome-wide significance level. Ingenuity pathway analysis suggested that genes predominantly involved in metabolic disease pathways were differentially methylated in women with gestational diabetes compared with those without [53]. In another study [54], DNA methylation levels at the maternally imprinted *MEST* gene were significantly lower in placenta and cord blood of women with gestational diabetes (88 treated dietetically and 98 with insulin) than in those without gestational diabetes. This study also found that morbidly obese adults had a decreased blood *MEST* methylation compared with normal-weight adults (*N* = 37 in each group). Animal models suggest that MEST plays a role in the development of obesity.

#### Fetal Epigenome and Long-Term Health

Several studies have examined associations of DNA methylation with later adiposity and cardiometabolic outcomes. In 1 study DNA methylation of the promoter regions of 5 candidate genes (*RXRA*, *eNOS*, *SOD1*, *IL8*, and *PI3KCD*) in umbilical cord tissue was related to dual X-ray absorptiometry (DXA)-determined fat mass in childhood, but only DNA methylation in *RXRA* was found to relate to later fat mass in the discovery (*N* = 78) and replication (*N* = 239) cohorts [55]. In another study, DNA methylation at 24 candidate gene sites in cord blood white cells were related to a range of anthropometric outcomes and differential methylation at 5 sites was associated with offspring BMI and/or fat mass at age 9 (*N* = 178), but none of these associations withstood adjustment for multiple statistical tests [56]. Lastly, in a study using 3 independent samples (*N* = 21, 107, and 154) of white blood cell collected in childhood or adolescence, DNA hypermethylation in intron 2 and 3 of *POMC*, a gene known to be related to greater BMI and obesity risk, was found to be associated with obesity [57]. In a separate sample, the pattern of DNA methylation in *POMC* in normal weight individuals was found to be stable between birth and 18 years, and was also the same as that found in the same sites in neurons of the hypothalamic arcuate nucleus obtained at postmortem from normal weight adults (*N* = 5). It was also found that the obesity related pattern of DNA hypermethylation was present prior to the development of obesity, leading the authors to conclude that the methylation patterns in *POMC* appears to occur early in embryogenesis, persist across life and is related to obesity.

Associations of variation in DNA methylation levels and exposures or outcomes of interest are susceptible to ‘classic’ confounding [58, 59]. Hence, associations of maternal gestational diabetes with DNA methylation and of the latter with adiposity and cardiometabolic health do not prove causality. Larger studies, further replication and the use of analytical approaches for determining causality and whether differential DNA methylation in offspring is indeed a mediator or not are important next steps in following up these highly interesting but tentative observations. Two-step epigenetic Mendelian randomization is one such analytical approach. As in a Mendelian randomization approach (described above), genetic variants are used as a proxy for an exposure of interest (here pregnancy diabetes) in step 1. In step 2, a genetic variant is used as a proxy for methylation and related to an outcome of interest (here long- term offspring adiposity, cardiometabolic outcomes or cognitive ability). Each step can be examined separately or both can be jointly interrogating thus, testing the entire pathway. This approach is not without its limitations (see [58, 59] for detailed discussions) but it also has the potential to significantly further our understanding of the putative role of DNA methylation as a mediator in associations of pregnancy diabetes and offspring long- term health.

## Conclusions

Current evidence supports a direct causal role for exposure to maternal diabetes in utero in determining offspring long- term greater adiposity and adverse cardiometabolic health. Whilst a majority of observational studies report associations of exposure to maternal pregnancy diabetes with lower cognitive ability, there is also evidence supporting an opposite ‘protective’ intrauterine effect of exposure to maternal pregnancy diabetes on offspring cognitive ability. Differential DNA methylation may play a role in mediating these effects but research in this area is still novel.

## References

[1] Bell R, Bailey K, Cresswell T, Hawthorne G, Critchley J (2008). Trends in prevalence and outcomes of pregnancy in women with pre-existing type I and type II diabetes. BJOG.

[2] de Andrés AL, Jiménez-García R, Carrasco-Garrido P (2012). Trends in pregestational diabetes among women delivering in Spain, 2001–2008. Int J Gynecol Obstet.

[3] Rajab KE, Issa AA, Hasan ZA, Rajab E, Jaradat AA (2012). Incidence of gestational diabetes mellitus in Bahrain from 2002 to 2010. Int J Gynecol Obstet.

[4] Buckley BS, Harreiter J, Damm P, Corcoy R, Chico A (2012). Gestational diabetes mellitus in Europe: prevalence, current screening practice and barriers to screening. A review. Diabet Med.

[5] Albrecht SS, Kuklina EV, Bansil P, Jamieson DJ, Whiteman MK (2010). Diabetes trends among delivery hospitalizations in the U.S., 1994–2004. Diabetes Care.

[6] National Institute for Health and Clinical Excellence (2008). NICE clinical guideline 63: diabetes in pregnancy: management of diabetes and its complications from preconception to the postnatal period.

[7] National Institute of Health. National Institutes of Health Consensus Development Conference on Diagnosing Gestational Diabetes Mellitus: Final Statement; 2013.

[8] Xiang AH, Li BH, Black MH, Sacks DA, Buchanan TA (2011). Racial and ethnic disparities in diabetes risk after gestational diabetes mellitus. Diabetologia.

[9] Association AD (2011). Diagnosis and classification of diabetes mellitus. Diabetes Care.

[10] Ogata ES (2010). Problems of the infant of the diabetic mother. NeoReviews.

[11] Hay W (2012). Care of the infant of the diabetic mother. Curr Diabet Rep.

[12.••] The HAPO Study Cooperative Research Group (2008). Hyperglycemia and adverse pregnancy outcomes. N Engl J Med.

[13] HAPO Study Cooperative Research Group (2009). Hyperglycemia and Adverse Pregnancy Outcome (HAPO) Study. Diabetes.

[14.•] Lawlor DA, The Society for Social Medicine John Pemberton lecture 2011 (2013). Developmental overnutrition—an old hypothesis with new importance?. Int J Epidemiol.

[15] Wood E (1936). Sugar content of mother’s blood after fasting: its relation to the birth weight of infants. Am J Dis Child.

[16] Whie PHH (1942). Pregnancy complicating diabetes: a report of clinical results. J Clin Endocrinol Metab.

[17] Sridhar SB, Ferrara A, Ehrlich SF, Brown SD, Hedderson MM (2013). Risk of large-for-gestational-age newborns in women with gestational diabetes by race and ethnicity and body mass index categories. Obstet Gynecol.

[18.•] Lawlor DA, Relton C, Sattar N, Nelson SM (2012). Maternal adiposity—a determinant of perinatal and offspring outcomes?. Nat Rev Endocrinol.

[19] Patel S, Fraser A, Davey Smith G, Lindsay RS, Sattar N (2012). Associations of gestational diabetes, existing diabetes, and glycosuria with offspring obesity and cardiometabolic outcomes. Diabetes Care.

[20] Gillman MW, Rifas-Shiman S, Berkey CS, Field AE, Colditz GA (2003). Maternal gestational diabetes, birth weight, and adolescent obesity. Pediatrics.

[21] Kim SY, England JL, Sharma JA, Njoroge T (2011). Gestational diabetes mellitus and risk of childhood overweight and obesity in offspring: a systematic review. Exp Diabetes Res.

[22] Philipps LH, Santhakumaran S, Gale C, Prior E, Logan KM (2011). The diabetic pregnancy and offspring BMI in childhood: a systematic review and meta-analysis. Diabetologia.

[23.•] Snell-Bergeon J, Dabelea D. The infant of the diabetic mother: metabolic imprinting. In: Tsatsoulis A, Wyckoff J, Brown FM, editors. Diabetes in women. NYC, NY: Humana Press; 2010; p. 359–75. *This is a thorough review of the long- term metabolic effects of exposure to pregnancy diabetes in utero and of studies in the Pima Indians in particular.*

[24] Pettitt DJ, Nelson RG, Saad MF, Bennett PH, Knowler WC (1993). Diabetes and obesity in the offspring of Pima Indian women with diabetes during pregnancy. Diabetes Care.

[25] Dabelea D (2000). Intrauterine exposure to diabetes conveys risks for type 2 diabetes and obesity: a study of discordant sibships. Diabetes.

[26] Lawlor DA, Lichtenstein P, Langstrom N (2011). Association of maternal diabetes mellitus in pregnancy with offspring adiposity into early adulthood: sibling study in a prospective cohort of 280,866 men from 248,293 families. Circulation.

[27] Rizzo TA, Dooley SL, Metzger BE, Cho NH, Ogata ES (1995). Prenatal and perinatal influences on long-term psychomotor development in offspring of diabetic mothers. Am J Obstet Gynecol.

[28] Nelson CA, Wewerka S, Thomas KM, Tribby-Walbridge S, deRegnier R (2000). Neurocognitive sequelae of infants of diabetic mothers. Behav Neurosci.

[29] Gin H, Vambergue A, Vasseur C, Rigalleau V, Dufour P (2006). Blood ketone monitoring: a comparison between gestational diabetes and nondiabetic pregnant women. Diabet Metab.

[30] Ornoy A, Wolf A, Ratzon N, Greenbaum C, Dulitzky M (1999). Neurodevelopmental outcome at early school age of children born to mothers with gestational diabetes. Arch Dis Child Fetal Neonatal.

[31] Ornoy A, Ratzon N, Greenbaum C, Peretz E, Soriano D (1998). Neurobehaviour of school age children born to diabetic mothers. Arch Dis Child Fetal Neonatal.

[32] Ornoy A, Ratzon N, Greenbaum C, Wolf A, Dulitzky M (2001). School-age children born to diabetic mothers and to mothers with gestational diabetes exhibit a high rate of inattention and fine and gross motor impairment. J Pediatr Endocrinol Metab.

[33] Nielsen GL, Dethlefsen C, Sørensen HT, Pedersen JF, Molsted-Pedersen L (2007). Cognitive function and army rejection rate in young adult male offspring of women with diabetes: a Danish population-based cohort study. Diabetes Care.

[34] Nomura Y, Marks DJ, Grossman B, Yoon M, Loudon H, Stone J, Halperin JM (2012). Exposure to gestational diabetes mellitus and low socioeconomic status: effects on neurocognitive development and risk of attention-deficit/hyperactivity disorder in offspring. Arch Pediatr Adolesc Med.

[35] Temple RC, Hardiman M, Pellegrini M, Horrocks L, Martinez-Cengotitabengoa MT (2011). Cognitive function in 6- to 12-year-old offspring of women with Type 1 diabetes. Diabet Med.

[36] Clausen TD, Mortensen EL, Schmidt L, Mathiesen ER, Hansen T (2011). Cognitive function in adult offspring of women with Type-á1 diabetes. Diabet Med.

[37] Clausen TD, Mortensen EL, Schmidt L, Mathiesen ER, Hansen T (2013). Cognitive function in adult offspring of women with gestational diabetes–the role of glucose and other factors. PLoS One.

[38] Dahlquist G, Kallen B (2007). School marks for Swedish children whose mothers had diabetes during pregnancy: a population-based study. Diabetologia.

[39] Fraser A, Nelson SM, Macdonald-Wallis C, Lawlor DA (2012). Associations of existing diabetes, gestational diabetes, and glycosuria with offspring IQ and educational attainment: the Avon Longitudinal Study of Parents and Children. Exp Diabetes Res.

[40] Veena SR, Krishnaveni GV, Srinivasan K, Kurpad AV, Muthayya S (2010). Childhood cognitive ability: relationship to gestational diabetes mellitus in India. Diabetologia.

[41] Rizzo T, Metzger BE, Burns WJ, Burns K (1991). Correlations between antepartum maternal metabolism and intelligence of offspring. N Engl J Med.

[42] Nielsen GL, Dethlefsen C, Sorensen HT, Pedersen JF, Molsted-Pedersen L (2007). Cognitive function and Army rejection rate in young adult male offspring of women with diabetes. Diabetes Care.

[43] Nielsen GL, Andersen E, Lundbye-Christensen S (2010). Maternal blood glucose in diabetic pregnancies and cognitive performance in offspring in young adulthood: a Danish cohort study. Diabet Med.

[44] Davey Smith G, Ebrahim S (2003). Mendelian randomization: can genetic epidemiology contribute to understanding environmental determinants of disease?. Int J Epidemiol.

[45] Lawlor DA, Harbord RM, Sterne JA, Timpson N, Davey SG (2008). Mendelian randomization: using genes as instruments for making causal inferences in epidemiology. Stat Med.

[46] Bonilla C, Lawlor D, Ben-Shlomo Y, Ness A, Gunnell D (2012). Maternal and offspring fasting glucose and type 2 diabetes-associated genetic variants and cognitive function at age 8: a Mendelian randomization study in the Avon Longitudinal Study of Parents and Children. BMC Med Genet.

[47] Fraser A, Almqvist C, Larsson H, Långström N, Lawlor D. Maternal diabetes in pregnancy and offspring cognitive ability: sibling study with 723,775 men from 579,857 families. Diabetologia. 2014;57:102–9. 10.1007/s00125-013-3065-z PMC3857877 24065154

[48] Jaenisch R, Bird A (2003). Epigenetic regulation of gene expression: how the genome integrates intrinsic and environmental signals. Nat Genet.

[49] Wong CC, Caspi A, Williams B, Craig IW, Houts R (2010). A longitudinal study of epigenetic variation in twins. Epigenetics.

[50] Day JJ, Sweatt JD (2012). Epigenetic treatments for cognitive impairments. Neuropsychopharmacology.

[51] Bouchard L (2010). Leptin gene epigenetic adaptation to impaired glucose metabolism during pregnancy. Diabetes Care.

[52] Bouchard L, Hivert MF, Guay SP, St-Pierre J, Perron P (2012). Placental adiponectin gene DNA methylation levels are associated with mothers’ blood glucose concentration. Diabetes.

[53] Ruchat SM, Houde AA, Voisin G, St-Pierre J, Perron P (2013). Gestational diabetes mellitus epigenetically affects genes predominantly involved in metabolic diseases. Epigenetics.

[54] El Hajj N, Pliushch G, Schneider E, Dittrich M, Müller T (2013). Metabolic programming of MEST DNA methylation by intrauterine exposure to gestational diabetes mellitus. Diabetes.

[55] Godfrey KM, Sheppard A, Gluckman PD, Lillycrop KA, Burdge GC (2011). Epigenetic gene promoter methylation at birth is associated with child’s later adiposity. Diabetes.

[56] Relton CL, Groom A, St. Pourcain B, Sayers AE, Swan DC (2012). DNA methylation patterns in cord blood DNA and body size in childhood. PLoS One.

[57] Kuehnen P, Mischke M, Wiegand S, Sers C, Horsthemke B (2012). An Alu element-associated hypermethylation variant of the POMC gene is associated with childhood obesity. PLoS Genet.

[58] Relton CL, Davey Smith G (2012). Two-step epigenetic Mendelian randomization: a strategy for establishing the causal role of epigenetic processes in pathways to disease. Int J Epidemiol.

[59] Relton CL, Davey Smith G (2010). Epigenetic epidemiology of common complex disease: prospects for prediction, prevention, and treatment. PLoS Med.

